# Optimizing strategies for population-based chlamydia infection screening among young women: an age-structured system dynamics approach

**DOI:** 10.1186/s12889-015-1975-z

**Published:** 2015-07-11

**Authors:** Yu Teng, Nan Kong, Wanzhu Tu

**Affiliations:** Futures Institute, 41-A New London Tpke, Glastonbury, Connecticut 06033 USA; Weldon School of Biomedical Engineering, Purdue University, 206 S. Martin Jischke Dr, West Lafayette, Indiana 47907 USA; Department of Biostatistics, Indiana University School of Medicine, 410 West 10th St, Suite 3000, Indianapolis, Indiana 46202 USA

**Keywords:** Age-structured system dynamics, Infectious disease modeling, Disease screening, Parameter optimization

## Abstract

**Background:**

Chlamydia infection (CT) is one of the most commonly reported sexually transmitted diseases. It is often referred to as a “silent” disease with the majority of infected people having no symptoms. Without early detection, it can progress to serious reproductive and other health problems. Economical identification of asymptomatically infected is a key public health challenge. Increasing evidence suggests that CT infection risk varies over the range of adolescence. Hence, age-dependent screening strategies with more frequent testing for certain age groups of higher risk may be cost-saving in controlling the disease.

**Methods:**

We study the optimization of age-dependent screening strategies for population-based chlamydia infection screening among young women. We develop an age-structured compartment model for CT natural progress, screening, and treatment. We apply parameter optimization on the resultant PDE-based system dynamical models with the objective of minimizing the total care spending, including screening and treatment costs during the program period and anticipated costs of treating the sequelae afterwards). For ease of practical implementation, we also search for the best screening initiation age for strategies with a constant screening frequency.

**Results:**

The optimal age-dependent strategies identified outperform the current CDC recommendations both in terms of total care spending and disease prevalence at the termination of the program. For example, the age-dependent strategy that allows monthly screening rate changes can save about 5 % of the total spending. Our results suggest early initiation of CT screening is likely beneficial to the cost saving and prevalence reduction. Finally, our results imply that the strategy design may not be sensitive to accurate quantification of the age-specific CT infection risk if screening initiation age and screening rate are the only decisions to make.

**Conclusions:**

Our research demonstrates the potential economic benefit of age-dependent screening strategy design for population-based screening programs. It also showcases the applicability of age-structured system dynamical modeling to infectious disease control with increasing evidence on the age differences in infection risk. The research can be further improved with consideration of the difference between first-time infection and reinfection, as well as population heterogeneity in sexual partnership.

## Background

Sexually transmitted infections with *Chlamydia trachomatis* (CT) are among the most commonly reported infectious diseases in the United States [10] and many other developed countries [38]. The infection is caused by bacterium *C. trachomatis* [7]. It is estimated that about 1 million individuals in the U.S. are infected with CT. Due to lack of specific symptoms in many CT infection cases [22], the infection may lead to major long-term morbidities such as pelvic inflammatory disease, ectopic pregnancy, and infertility [9, 36]. Together with other STDs, CT infection inflicts significant human and economic costs [26].

At present, CT infection can be accurately detected and easily treated with early detection. Thus, CT screening has emerged as a key public health intervention [6] and the disease control relies primarily on the cost and effectiveness of the screening. Several economic studies found CT screening to be cost-effective, and even cost-saving (e.g., [17–19, 21, 35]). For literature reviews on the economic studies, we refer to Low et al. [23, 24]; Roberts et al. [28]. However, most of the existing economic studies assumed a constant CT infection rate over the studied age range, which typically spans adolescence and early adulthood. Increasing evidence suggests that the CT infection risk decreases with age (e.g., [3, 13, 31]), mainly due to more stabilized sexual partnership and possibly also due to increased immunological response to CT over age. Hence, one would expect that a screening strategy with age-dependent screening rate, i.e., treating screening proportion in the population as a function of age, would be more cost-saving than the strategies assuming a constant rate. In this paper, we incorporate the age dependency of the infection risk into an economic study of CT screening with nucleic acid amplification testing [33]. We optimize age-dependent screening strategies for a population-based screening program, which offers tests systematically to all individuals in the target group within a framework of agreed policy, protocols, quality management, monitoring and evaluation [16].

To the best of our knowledge, only few simulation-based economic studies have taken the age-dependency into account. For example, Hu et al. [18, 19], basing their studies on an earlier observational study in the Netherlands [8, 15], assumed that the probability of acquiring CT is constant for women from early ages and decreases with a constant annual rate after then. While the simulation-based analyses have compared tailored screening strategies that recommend different screening rates to different population subgroups based on some risk measure (e.g., [18, 19, 21]), we have not witnessed any optimization work on identifying age-dependent CT screening strategies, which are, in some sense, a subset of risk-based strategies.

In this paper, we model the population dynamics, related to CT transmission, screening, and treatment, with a set of partial differential equations (PDE) that incorporate age-dependency on the CT infection risk. We formulate a parameter optimization problem subject to the PDE model to identify the screening rates at different age points over a range (i.e., an age-dependent parameter profile) such that some per-capita cumulative cost is minimized. To summarize our contribution, we are among the first that conduct economic analyses of population-based CT screening programs through age-structured systems modeling and optimization.

In this paper, we also reasonably specify the studied cohort so that we can reduce the PDE model to a set of ordinary differential equations (ODEs) for simplifying the numerical optimization. We next focus on the optimization over a set of more implementable strategies. In anticipation that the optimal age-specific screening strategy may be difficult to implement as optimal screening rates obtained from the above model may vary significantly between consecutive age points, we consider cases where a constant screening rate is applied to a truncated age range. Specifically, we consider optimizing the screening start age. Finally, we make a simplifying assumption on the age-specific infection risk, with which we remodel the system dynamics and explore the benefit in the numerical optimization. Through this simplification, we also check how robust the optimal strategy with a constant screening rate is to the estimate of the age-specific CT infection risk profile. After presenting the research methodology, we report our numerical studies and discuss their policy implications. At the end of the paper, we draw conclusions and outline future research.

Differential equation based systems dynamic modeling has been widely used in infectious disease control. For a general introduction, we refer to Keeling and Rohani [20]. For studies on CT transmission dynamics, we refer to Martin et al. [25]; Sharomi and Gumel [29]. Meanwhile, ODE-based models have been applied to economic studies of screening programs. For example, Althaus et al. [1] applied an SEIRS (susceptible-exposed-infected-recovered-susceptible) model, which is widely used in the infectious disease modeling literature (e.g., [2, 15]), to assess the impact of screening programs on CT prevalence reduction. Regan et al. [27] extended the SEIRS model to incorporate the additional state of receiving treatment. Note that the two studies above did not consider cost or cost-effectiveness of the screening programs. Our work differs from previous in that we apply nonlinear optimization to design optimal strategies.

## Methods

### Optimization of age-dependent screening strategies

#### An age-structured SEIRS model

We adapt a widely used SEIRS compartment model [1] to illustrate the system dynamics associated with CT transmission, screening, and treatment. We then capture the system dynamics with a multi-compartment model and mathematically formalize the age-structured population heterogeneity with a set of PDEs.

Compartment modeling has been widely used in modeling infectious disease transmission [2, 4, 20, 34]. In recent years, it has been used to model various specific screening, vaccinating, pharmaceutical, and therapeutic interventions for dealing with relevant public health problems (e.g., [12]). To many infectious diseases, age has a deep influence on the rate of disease spread in a population, especially the contact rate [2, 20]. To sexually transmitted diseases, the contact rate is affected by the sexual behavior, which is often age dependent.

**Fig. 1:**
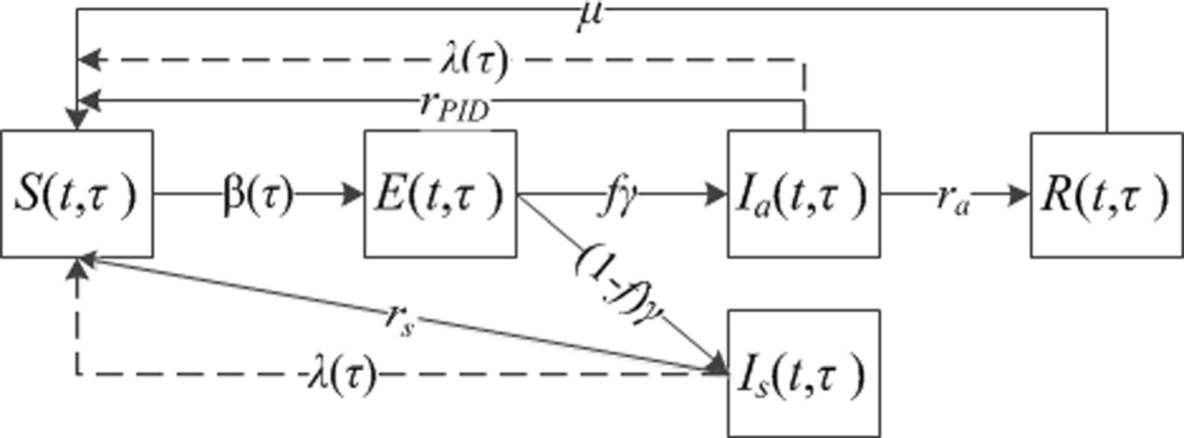
An age-dependent SIER Model for CT transmission and screening. Each box (compartment) represents a particular state that the total population is stratified into. For instance, *S* standards for the susceptible population subgroup. The solid lines indicate transitions due to natural disease progression and standard therapeutic intervention; and the dashed lines indicate additional transitions due to screening. With the system dynamics, each subpopulation size may fluctuate over time. Note that this is an age-structured model, which implies that the fluctuation of each subpopulation size is also age dependent, i.e.,many transition rates are age-dependent such as *β*

Figure 1 presents the age-structured compartment model. In the figure, the solid lines indicate transitions following the natural history and standard pharmaceutical/therapeutic intervention of the disease. The dashed lines indicate additional transitions due to screening. The system dynamics is explained as follows. Let *t* and *τ* be the time and age indices, respectively. At any time *t ε* [0, *T*], each population of age *τ ε* [0, *A*] is divided into five subgroups as follows. Susceptible population subgroup, denoted by *S*(*t*,*τ*), infected by the entire infected population with an age-dependent rate *β*(*τ*) > 0. They then experience an incubation period at rate *γ* > 0, during which they are denoted by *E*(*t*,*τ*). After the incubation period, the infection symptom becomes onset among a fraction of the infected population, denoted by *I*_*s*_(*t*,*τ*), whereas other infected people, denoted by *I*_*a*_(*t*,*τ*), do not show any symptom. We denote *f ε* [0, 1] to be the probability that an infected individual remains asymptomatic. In the absence of screening, symptomatically infected people clear their infections at a rate *r*_*s*_ > 0, which can be interpreted as treating the infection by a general practitioner with symptom onset and subsequently curing the disease. We assume that the treatment is sought immediately after the symptom onset. Asymptomatically infected people may develop acute pelvic inflammatory disease (PID), then immediately seek inpatient treatment, and subsequently cure the disease at a rate *r*_*PID*_ >0. Alternatively, they may recover through natural clearance a rate *r*_*a*_ >0. We denote such people to be *R*(*t*,*τ*) and denote *μ* > 0 to be the rate at which they have temporary immunity before becoming susceptible to reinfection. With screening, the entire population is screened at an age-specific rate *λ*(*τ*) (i.e., on average each individual will be screened within 1/ *λ*(*τ*) years from age point *τ*). We assume the screening test is 100 % accurate and treatment is sought immediately after an infection is detected. We further assume that the screening is independent of the processes of infection clearance among both asymptomatically and symptomatically infected people. Hence, with screening, the overall infection clearance rates are *r*_*s*_ + *λ*(*τ*) and *r*_*PID*_ + *λ*(*τ*) for symptomatically and asymptomatically infected, respectively.

The notation used in the model is summarized in Table 1. The system dynamics is described with the following PDEs. In mathematics, a PDE is a differential equation that contains unknown multivariate functions and their partial derivatives. It is in contrast to ordinary differential equations (ODEs), which deal with functions of a single variable and their derivatives. PDEs are used to formulate problems involving functions of multiple variables. PDEs can be used to describe a wide variety of phenomena such as sound, heat, electrostatics, electrodynamics, fluid flow, elasticity, or quantum mechanics. For a general introduction on PDE, we refer to [14].

**Table 1:** Notation in the age-structured compartment model and corresponding PDEs

*S*(*t*,*τ*)	Susceptible	*f*	Fraction of asymptomatic infections
*E*(*t*,*τ*)	Exposed	1/*γ*	Incubation time
*I*_*a*_(*t*,*τ*)	Asymptomatically infected	1/*r*_*a*_	Duration of the asymptomatic period
*I*_*s*_(*t*,*τ*)	Symptomatically infected	1/*r*_*s*_	Duration of the symptomatic period
*R(t*,*τ*)	Recovered	1/*μ*	Duration of the temporary immunity
β(*τ*)	Age-dependent infection rate		after natural clearance of asymptomatic infection
*λ*(*τ*)	Age-dependent screening rate	1/*r*_*PID*_	Duration of acute PID onset

$$ \begin{array}{l}\left(\frac{\partial }{\partial t}+\frac{\partial }{\partial \tau}\right)S\left(t,\tau \right)=-\beta \left(\tau \right)S\left(t,\tau \right){\displaystyle \underset{0}{\overset{A}{\int }}\left({I}_a\left(t,{\tau}^{\hbox{'}}\right)+{I}_s\left(t,{\tau}^{\hbox{'}}\right)\right)}d{\tau}^{\hbox{'}}+\left({r}_{PID}+\lambda \left(\tau \right)\right){I}_a\left(t,\tau \right)+\left({r}_s+\lambda \left(\tau \right)\right){I}_s\left(t,\tau \right)+\mu R\left(t,\tau \right);\hfill \\ {}\left(\frac{\partial }{\partial t}+\frac{\partial }{\partial \tau}\right)E\left(t,\tau \right)=\beta \left(\tau \right)S\left(t,\tau \right){\displaystyle \underset{0}{\overset{A}{\int }}\left({I}_a\left(t,{\tau}^{\hbox{'}}\right)+{I}_s\left(t,{\tau}^{\hbox{'}}\right)\right)}d{\tau}^{\hbox{'}}-\gamma E\left(t,\tau \right);\hfill \\ {}\left(\frac{\partial }{\partial t}+\frac{\partial }{\partial \tau}\right){I}_a\left(t,\tau \right)=f\gamma E\left(t,\tau \right)-\left({r}_a+{r}_{PID}+\lambda \left(\tau \right)\right){I}_a\left(t,\tau \right);\hfill \\ {}\left(\frac{\partial }{\partial t}+\frac{\partial }{\partial \tau}\right){I}_s\left(t,\tau \right)=\left(1-f\right)\gamma E\left(t,\tau \right)-\left({r}_s+\lambda \left(\tau \right)\right){I}_s\left(t,\tau \right);\hfill \\ {}\left(\frac{\partial }{\partial t}+\frac{\partial }{\partial \tau}\right)R\left(t,\tau \right)={r}_a{I}_a\left(t,\tau \right)-\mu R\left(t,\tau \right).\hfill \end{array} $$ Typically, a screening program estimates in advance the size of the cohort it can deal with based on its capacity and keeps its size relatively constant by synchronizing the recruitment and exit processes. Without loss of generality, we set the cohort size to be 1 at any time point, i.e., ${\displaystyle \underset{0}{\overset{A}{\int }}\left(S\left(t,\tau \right)+E\left(t,\tau \right)+{I}_a\left(t,\tau \right)+{I}_s\left(t,\tau \right)+R\left(t,\tau \right)\right)}d\tau =1,\forall t.$

Once the screening rate profile, as well as the boundary and initial conditions, are given, the state of the system can be determined for any given time point with the above PDEs. The screening and treatment costs are cumulated accordingly over the program duration. An optimal screening rate profile can then be identified to minimize the per-capita cumulative cost. We next present a parameter optimization problem subject to the PDE constraints.

#### A parameter optimization problem

We present an optimal screening strategy design problem for the generic screening program. In the objective function, we denote *c*_*s*_ to be the unit-time cost of screening an individual for CT, *c*_*t*_ to be the unit-time cost of treating an individual for CT with antibiotics, *c*_*PID*_ to be the unit-time cost of treating an individual for acute PID, and *c*_*end*_ to be the expected cost of treating an individual for possible future PID sequelae when she leaves the cohort at age *A* with undiagnosed asymptomatic CT. The expectation takes the probability of developing three major PID sequelae (i.e., chronic pelvic pain, ectopic pregnancy, and infertility) and their associated treatment cost. Given a screening rate profile *λ*(*τ*), we model four types of cumulative cost over a screening period of *T* as follows.

- CT screening cost: ${C}_s\left(\lambda \left(\tau \right)\right)={c}_sT{\displaystyle \underset{0}{\overset{A}{\int }}\lambda \left(\tau \right)},$
- CT Treatment cost: ${C}_t\left(\lambda \left(\tau \right)\right)={\displaystyle \underset{0}{\overset{T}{\int }}{\displaystyle \underset{0}{\overset{A}{\int }}{c}_t\left[\left({r}_s+\lambda \left(\tau \right)\right){I}_s\left(t,\tau \right)+\lambda \left(\tau \right){I}_a\left(t,\tau \right)\right]}} d\tau dt,$
- Acute PID treatment cost: ${C}_{PID}\left(\lambda \left(\tau \right)\right)={\displaystyle \underset{0}{\overset{T}{\int }}{\displaystyle \underset{0}{\overset{A}{\int }}{c}_{PID}}}{r}_{PID}{I}_a\left(t,\tau \right) d\tau dt,$
- PID sequelae treatment cost: ${C}_{end}\left(\lambda \left(\tau \right)\right)={\displaystyle \underset{0}{\overset{T}{\int }}{c}_{end}{I}_a\left(t,A\right)dt}.$

Note that the screening cost applies to the entire cohort, which is assumed to be 1. We define the cumulative cost as *C*_*total*_(*λ*(*τ*)) = *C*_*s*_(*λ*(*τ*)) + *C*_*t*_(*λ*(*τ*)) + *C*_*PID*_(*λ*(*τ*)) + *C*_*end*_(*λ*(*τ*)). The optimization problem is then formulated as $\underset{\lambda \left(\tau \right)}{ \min }{C}_{total}\left(\lambda \left(\tau \right)\right)$ subject to the PDEs introduced above and the boundary and initial conditions. While attempting to minimize the per-capita cumulative cost, we also compare different strategies in terms of the terminal CT prevalence at time *t*, defined as ${\displaystyle \underset{0}{\overset{A}{\int }}\left({I}_a\left(t,\tau \right)+{I}_s\left(t,\tau \right)\right)d\tau }$.

To solve this parameter optimization problem, we discretize it to a finite-dimensional nonlinear programming problem. Note that the discretization does not significantly affect the solution quality given that 1) many model parameters have only age-dependent point estimates; and 2) it is not feasible to modify the screening intensity in a continuous fashion. We divide the time interval [0, *T*) into *N*_*t*_ subintervals with equal step size *h*_*t*_, i.e., *N*_*t*_*h*_*t*_ = *T*. Then the end points of the subintervals are ${t}_0=0,{t}_1={h}_t,\dots, {t}_{N_t}=T$. We divide the age interval [0, *A*) into *N*_*τ*_ subintervals with equal step size *h*_*τ*_. Then the end points of the subintervals are ${\tau}_0=0,{\tau}_1={h}_{\tau },\dots, {t}_{N_{\tau }}=A$. We use *i*, *j* to denote the indices for time and age, respectively. We use *β*^*j*^ and *λ*^*j*^ to denote the discretized values for *β*(*τ*) and *λ*(*τ*) with *τ* = *jh*_*A*_. The PDEs for each *i* = 0, …, *N*_*T*_ - 1, and *j* = 0, …, *N*_*τ*_ - 1, are then discretized as follows.

$$ \begin{array}{l}\frac{S^{i+1,j}-{S}^{i,j}}{h_t}+\frac{S^{i,j+1}-{S}^{i,j}}{h_{\tau }}=-{\beta}^j{S}^{i,j}{\displaystyle \sum_{k=0}^{N_{\tau }-1}\left({I}_a^{i,k}+{I}_s^{i,k}\right)}+\left({r}_{PID}+{\lambda}^j\right){I}_a^{i,j}+\left({r}_s+{\lambda}^j\right){I}_s^{i,j}+\mu {R}^{i,j};\\ {}\frac{E^{i+1,j}-{E}^{i,j}}{h_t}+\frac{E^{i,j+1}-{E}^{i,j}}{h_{\tau }}={\beta}^j{S}^{i,j}{\displaystyle \sum_{k=0}^{N\tau -1}\left({I}_a^{i,k}+{I}_s^{i,k}\right)}-\gamma {E}^{i,j};\\ {}\frac{I_a^{i+1,j}-{I}_a^{i,j}}{h_t}+\frac{I_a^{i,j+1}-{I}_a^{i,j}}{h_{\tau }}=f\gamma {E}^{i,j}-\left({r}_a+{r}_{PID}+{\lambda}^j\right){I}_a^{i,j};\\ {}\frac{I_s^{i+1,j}-{I}_s^{i,j}}{h_t}+\frac{I_s^{i,j+1}-{I}_s^{i,j}}{h_{\tau }}=\left(1-f\right)\gamma {E}^{i,j}-\left({r}_s+{\lambda}^j\right){I}_s^{i,j};\\ {}\frac{R^{i+1,j}-{R}^{i,j}}{h_t}+\frac{R^{i,j+1}-{R}^{i,j}}{h_{\tau }}={r}_a{I}_a^{i,j}-\mu {R}^{i,j}.\end{array} $$

The objective function is discretized as: ${C}_{total}\left({\lambda}^0,{\lambda}^1,\dots, {\lambda}^{N_{\tau }-1}\right)={c}_sT{\displaystyle \sum_{j=0}^{N_{\tau }-1}{\lambda}^j}+{\displaystyle \sum_{i=0}^{N_T-1}{\displaystyle \sum_{j=0}^{N_{\tau }-1}{c}_t\left[\left({r}_s+{\lambda}^{i,j}\right){I}_s^{i,j}+{\lambda}^{i,j}{I}_a^{i,j}\right]}+}{\displaystyle \sum_{i=0}^{N_T-1}{\displaystyle \sum_{j=0}^{N_{\tau }-1}{c}_{PID}{r}_{PID}{I}_a^{i,j}}+}{\displaystyle \sum_{i=0}^{N_T-1}{c}_{end}{I}_a^{i,{N}_{\tau }}}.$

Given the two subinterval counts (*N*_*t*_ and *N*_*τ*_), the boundary and initial conditions, and the estimated CT infection risk for *j* = 1,…, *N*_*τ*_, we obtain a nonlinear optimization model with finitely many decision variables, linear objective function, and quadratic constraints. We use standard constrained nonlinear optimization solvers (e.g., active-set and interior point) available in the MATLAB Optimization Toolbox [4].

#### A special case for cohorts with uniform age distribution

In this section, we consider a special case of the above PDE model, which is more suitable to the real practice of a screening program. In real practice, a screening program often only targets those of age 0 (i.e., the smallest age to be concerned for CT infection) for recruitment and terminates CT screening for those who reach *A* (i.e., the largest age to be concerned for CT infection). A general belief is that the number of infected individuals at age 0 is negligible. That is, for any *t*, we have *S*(*t*,0) = *p*, where *p* is denoted as the rate with which new participants enter the cohort, and *E*(*t*,0) = *I*_*a*_(*t*,0) = *I*_*s*_(*t*,0) = *R*(*t*,0) = 0. We further assume that the age of the studied open cohort follows a uniform distribution and term such a cohort *uniformly aged cohort*. That is, for any *t*, we have *S*(*t*,*τ*) + *E*(*t*,*τ*) + *I*_*a*_(*t*,*τ*) + *I*_*s*_(*t*,*τ*) + *R*(*t*,*τ*) = *p* = 1/*A* for *τ* ∈ (0, *A*]. Hence, we can align the age domain with the time domain and thus reduce the age-structured PDE model to a time-invariant ODE model with age-specific CT infection risks. We term this model ODE_1. Since the screening strategy design is only considered up to age *A*, *β*(*τ*) for *τ* ≥ *A* can be arbitrarily specified. To solve the parameter optimization problem for ODE_1, we again resort to discretization. In the following, we further study this special case with a smaller set of age-independent screening strategies, which are more implementable in practice.

### Optimization of age-independent screening policies

Our study in this section was inspired by the current CT screening recommendations. The CDC guideline recommends annual CT screening for women under age 25 but does not specify the initial screening age [34]. We consider policies similar to the current CDC recommendations structure-wise. The considered policies recommend to start CT screening for women at some age between 0 and *A*, and continue the screening until *A* with a constant frequency. Hence, the optimization problem is intended to determine an optimal screening initiation age and optimal screening rate. Note that Teng et al. [12] studied the problem with fixed screening initiation age and only optimized the screening rate over a fixed age range. Their problem is a parameter optimization problem with only one decision variable and assumes a constant infection risk. For each screening initiation age $\widehat{\tau}$, we have a similar parameter optimization problem, but with age-dependent infection risk. We use a standard line search algorithm without derivative information in MATLAB to solve the inner problem for each given screening initiation age. We apply one-dimensional explicit enumeration to select the optimal screening initiation age.

**Fig. 2:**
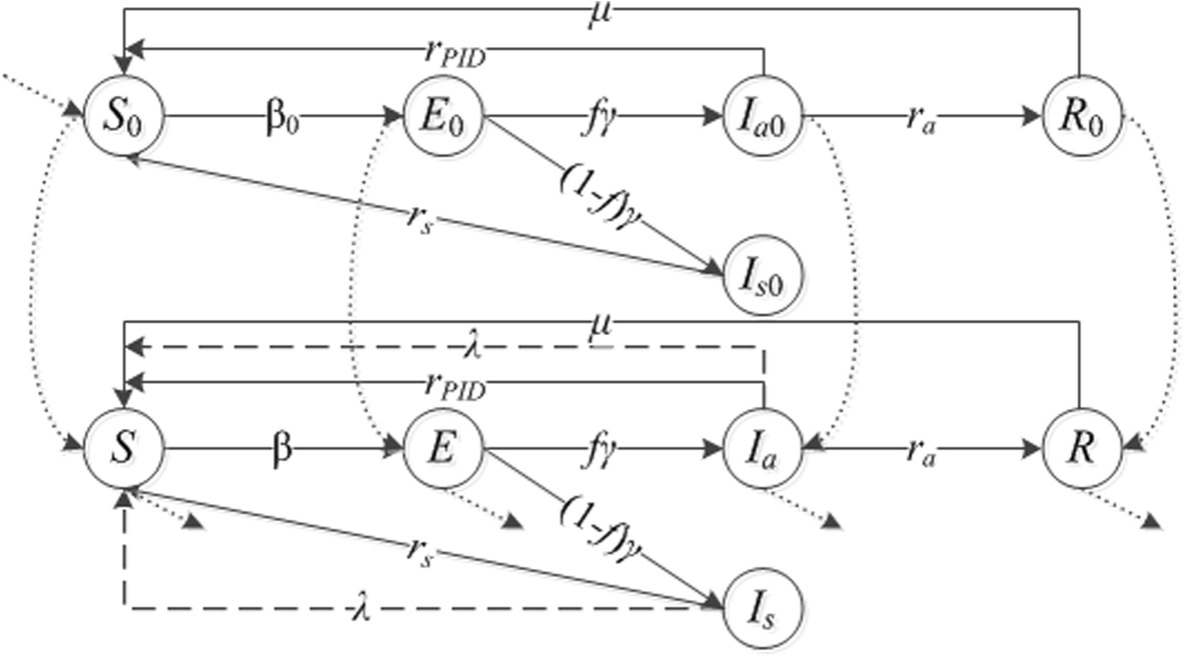
An age-independent SIER Model with two constant CT infection rates over the periods before and after screening initiation. This is a 10-compartment model with two portions. The upper portion captures the disease progression without screening from age 0 to the age determined to start screening. The lower portion captures the disease progression with screening from the age determined to start screening to age *A.* The solid lines and dashed lines are used in the same way as in Fig. 1 to indicate the dynamics. The dotted lines indicate the necessary vital dynamics with population aging

We further our study on this set of age-independent screening policies by considering a simplified case where the CT infection risk is assumed to be constant within the interval before screening initiation and within the interval after the initiation, respectively. With this simplification, the CT dynamical system is approximated with a two-part age-independent time-invariant coupling systems. We expect that solving the optimization problem on the two-part coupling system could decrease the computational time while only suffering slight reduction in terms of solution quality. Figure 2 illustrates a 10-compartment model for the two-part system. Given screening initiation age $\widehat{\tau}$, we divide the interval [0, *A*) into two subintervals [0, $\widehat{\tau}$) and [$\widehat{\tau}$, *A*). We use *S*_0_, *E*_0_, *I*_*a*0_, *I*_*s*0_, and *R*_0_ to denote the compartments for age range [0, $\widehat{\tau}$), and use *S*, *E*, *I*_*a*_, *I*_*s*_, and *R* to denote the compartments for age range [$\widehat{\tau}$, *A*). The disease transmission occurs in both age ranges, while screening is administered only to [$\widehat{\tau}$, *A*). With the assumption of two constant CT infection risks, we use *β*_0_ and *β* to denote the risks in the two age ranges, respectively. All cost and other transition parameters remain the same as introduced earlier.

To formulate the optimization problem, we denote *M*_0_ and *M* to be the total populations in the two age ranges. With an uniformly aged cohort, we have *M*_0_/*M* = $\widehat{\tau}$/(*A* - $\widehat{\tau}$) and *M*_0_ + *M* = 1 for any given $\widehat{\tau}$. We can thus uniquely determine the values of *M*_0_ and *M*. With the above notation, we introduce model ODE_2 as follows. For [0, $\widehat{\tau}$), the system dynamics is governed by

$$ \begin{array}{l}\frac{d{S}_0}{d\tau }=-{\beta}_0{S}_0\frac{\left({I}_{a0}+{I}_{s0}\right)}{M_0}+{r}_{PID}{I}_{a0}+{r}_s{I}_{s0}+\mu {R}_0+p-\frac{S_0}{M_0}p;\\ {}\frac{d{E}_0}{d\tau }={\beta}_0{S}_0\frac{\left({I}_{a0}+{I}_{s0}\right)}{M_0}-\gamma {E}_0-\frac{E_0}{M_0}p;\\ {}\frac{d{I}_{a0}}{d\tau }=f\gamma {E}_0-\left({r}_{PID}+{r}_a\right){I}_{a0}-\frac{I_{a0}}{M_0}p;\\ {}\frac{d{I}_{s0}}{d\tau }=\left(1-f\right)\gamma {E}_0-{r}_s{I}_{s0}-\frac{I_{s0}}{M_0}p;\\ {}\frac{d{R}_0}{d\tau }={r}_a{I}_{a0}-\mu {R}_0-\frac{R_0}{M_0}p.\end{array} $$

For age range [$\widehat{\tau}$, *A*), the system dynamics is governed by

$$ \begin{array}{l}\frac{dS}{d\tau }=-\beta S\frac{\left({I}_a+{I}_s\right)}{M}+\left({r}_{PID}+\lambda \right){I}_a+\left({r}_s+\lambda \right){I}_s+\mu R+\frac{S_0}{M_0}p-\frac{S}{M}p;\\ {}\frac{dE}{d\tau }=\beta S\frac{\left({I}_a+{I}_s\right)}{M}-\gamma E+\frac{E_0}{M_0}p-\frac{E}{M}p;\\ {}\frac{d{I}_a}{d\tau }=f\gamma E-\left({r}_{PID}+{r}_a+\lambda \right){I}_a+\frac{I_{a0}}{M_0}p-\frac{I_a}{M}p;\\ {}\frac{d{I}_s}{d\tau }=\left(1-f\right)\gamma E-\left({r}_s+\lambda \right){I}_s+\frac{I_{s0}}{M_0}p-\frac{I_s}{M}p;\\ {}\frac{dR}{d\tau }={r}_a{I}_a-\mu R+\frac{R_0}{M_0}p-\frac{R}{M}p.\end{array} $$

We present the objective function with respect to the screening initiation age $\widehat{\tau}$ and constant screening rate *λ*.

- CT screening cost: ${C}_s\left(\widehat{\tau},\lambda \right)={c}_s\lambda M\left(A-\widehat{\tau}\right),$
- CT treatment cost: ${C}_t\left(\widehat{\tau},\lambda \right)={\displaystyle \underset{0}{\overset{A}{\int }}{c}_t\left[{r}_s\left({I}_{s0}+{I}_s\right)+\lambda \left({I}_s+{I}_a\right)\right]d\tau },$
- Acute PID treatment cost: ${C}_{PID}\left(\widehat{\tau},\lambda \right)={\displaystyle \underset{0}{\overset{A}{\int }}{c}_{PID}{r}_{PID}\left({I}_{a0}+{I}_a\right)d\tau },$
- PID sequelae treatment cost: ${C}_{end}\left(\widehat{\tau},\lambda \right)={\displaystyle \underset{0}{\overset{A}{\int }}{c}_{end}p\frac{I_a}{M}d\tau },$
- Per-capita cumulative cost: ${C}_{total}\left(\widehat{\tau},\lambda \right)={C}_s\left(\widehat{\tau},\lambda \right)+{C}_t\left(\widehat{\tau},\lambda \right)+{C}_{PID}\left(\widehat{\tau},\lambda \right)+{C}_{end}\left(\widehat{\tau},\lambda \right).$

The optimization problem is thus presented as $\underset{\widehat{\tau},\lambda }{ \min }{C}_{total}\left(\widehat{\tau},\lambda \right)$ subject to ODE_2.

With any given screening initiation age $\widehat{\tau}\in \left[0,A\right)$, *β*_0_ and *β* become known. Hence, we can uniquely set the initial condition on *S*($\widehat{\tau}$), *E*($\widehat{\tau}$), *I*_*a*_($\widehat{\tau}$), *I*_*s*_($\widehat{\tau}$), and *R*($\widehat{\tau}$). We also determine the cost accumulated from 0 to $\widehat{\tau}$. Then we can reduce the optimization problem to a parameter optimization problem based on the 5-compartment ODE model for $\tau \in \left[\widehat{\tau},A\right)$, for which we can adapt the optimization method proposed in Teng et al. [12]. That is, for any $\widehat{\tau}$, the gradient of the objective function, i.e., $\frac{d{C}_{total}\left(\widehat{\tau},\lambda \right)}{d\lambda }$, can be derived with a cubic interpolation method. We apply a standard linear search algorithm with derivatives in MATLAB to solve the inner problem given each screening initiation age. We then apply one-dimensional explicit enumeration to select the optimal screening initiation age.

## Results and discussion

**Fig. 3:**
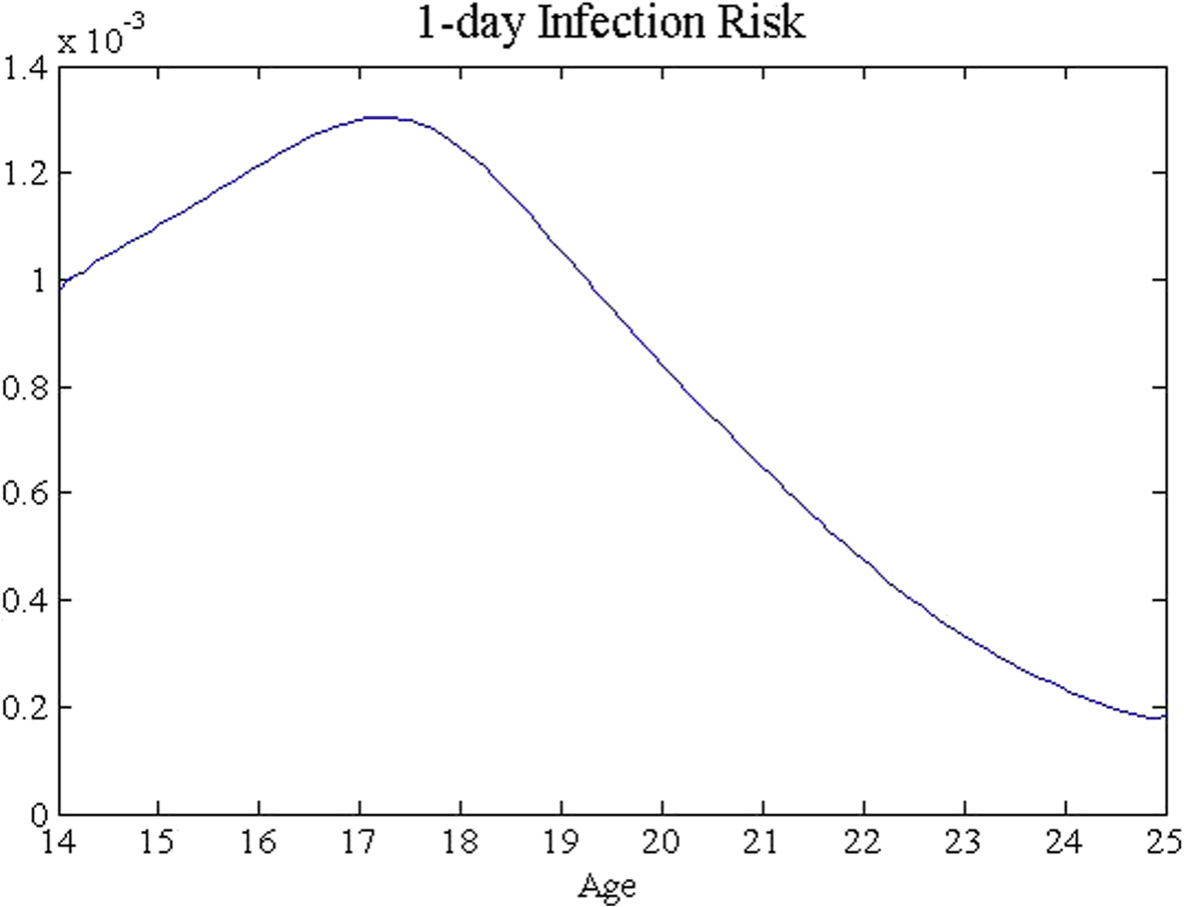
Initial condition for model ODE_1

We focus on the special case of uniformly aged cohort for our proof-of-concept numerical studies. We acquired model parameters from [1, 18] (Table 2). We estimated age-dependent infection risk *β*(*τ*) based on a longitudinal study of CT infection among recruited inter-city young women in a Midwest U.S. city [14] (Fig. 3). We set the screening initiation and termination ages to be 14 and 25, respectively, largely according to the CDC recommendation on the universal screening. Note that some of the work in the existing literature has conducted economic studies on annual and biannual universal screening beyond age 25. It is clear that we can extend the upper bound of the integrations (i.e., increase *A*) to accommodate this change. We will leave it to our future study.

**Table 2:** Parameters pertaining to costs and disease transition rates

	Parameter value
*f*	0.625
1/*γ*	14 days
1/*r*_*a*_	433 days
1/*r*_*s*_	35 days
1/*μ*	90 days
1/*r*_*PID*_	1000 days
*c*_*s*_	$13
*c*_*t*_	$36
*c*_*PID*_	$1898
*c*_*end*_	$192

We study the three parameter optimization problems presented earlier. In summary, the first problem aims to identify an optimal age-dependent screening strategy based on the time-invariant ODE model with an age-specific CT infection risk profile (i.e., ODE_1). The second problem aims to identify the screening initiation age and constant screening rate thereafter, again based on ODE_1. The third problem aims to make the same set of decisions as the second problem but the problem is based on the two-part time-invariant ODE model with a constant CT infection risk over each of the two age ranges (i.e., ODE_2). We term the optimal screening strategies identified in the three optimization problems S1, S2, and S3 in that order. We compare the three optimal strategies both in per-capita cumulative cost and terminal CT prevalence. We also report a comparative study with no screening and with the current recommendations.

**Fig. 4:**
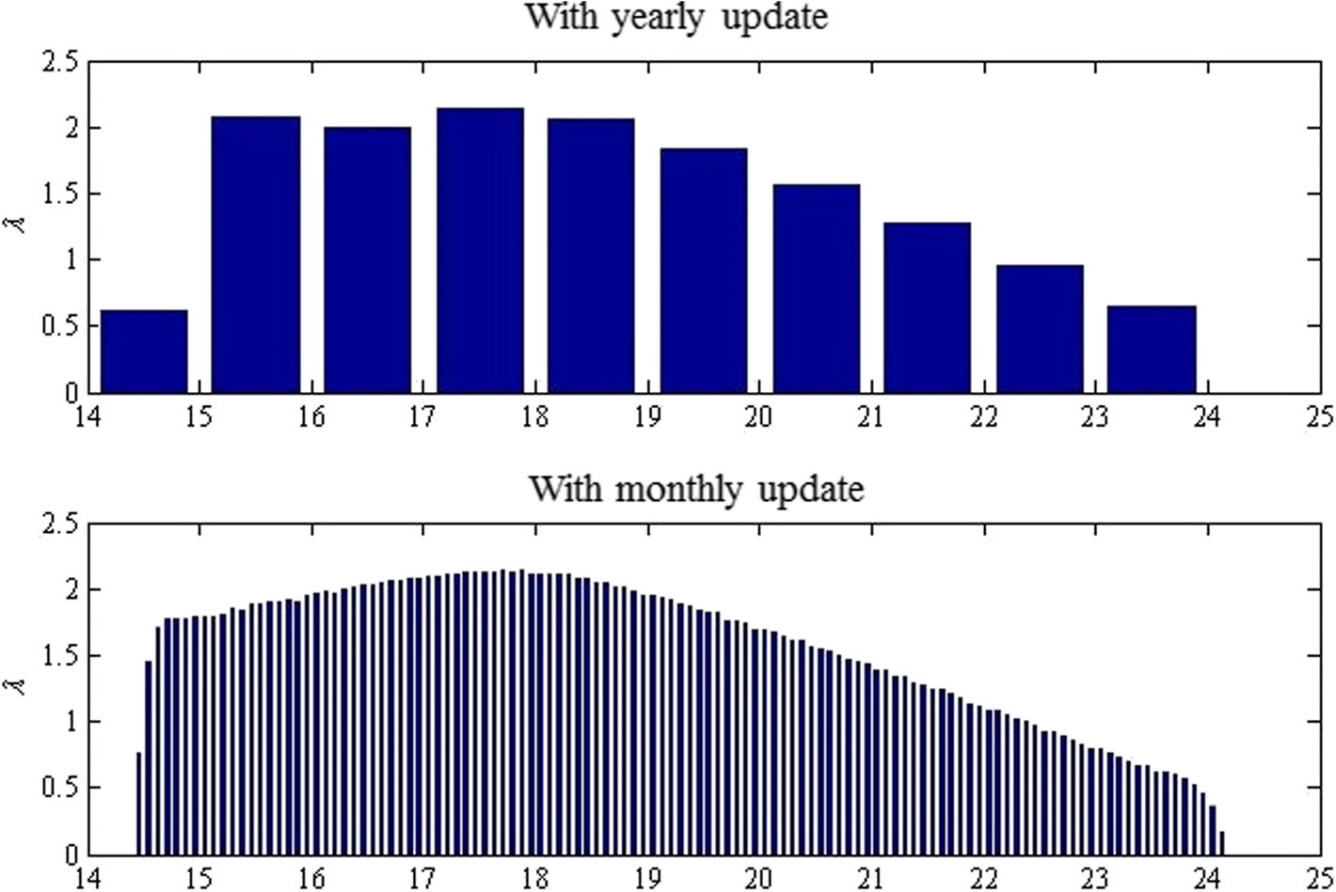
Optimal age-dependent screening strategy (S1)
- For S2, we present the optimal screening rate with all possible screening initiation ages (every month between 0 and *A*), as well as the associated per-capita cumulative cost and terminal prevalence in Fig. 5. The smallest unit for the screening initiation age is one month. The strategy with the minimum cost is the one that starts the screening for every individual when she reaches the 6^th^ month after the 14^th^ birthday. The screening rate is 1.511 times per year, which implies that an individual should test for CT roughly every 8 months.

- For S1, the screening rate profile is represented as a multi-step function with identical step size depending on the maximal allowable frequency of strategy update. We chose to update the screening strategy either yearly or monthly. We report the optimal strategies in Fig. 4.

**Fig. 5:**
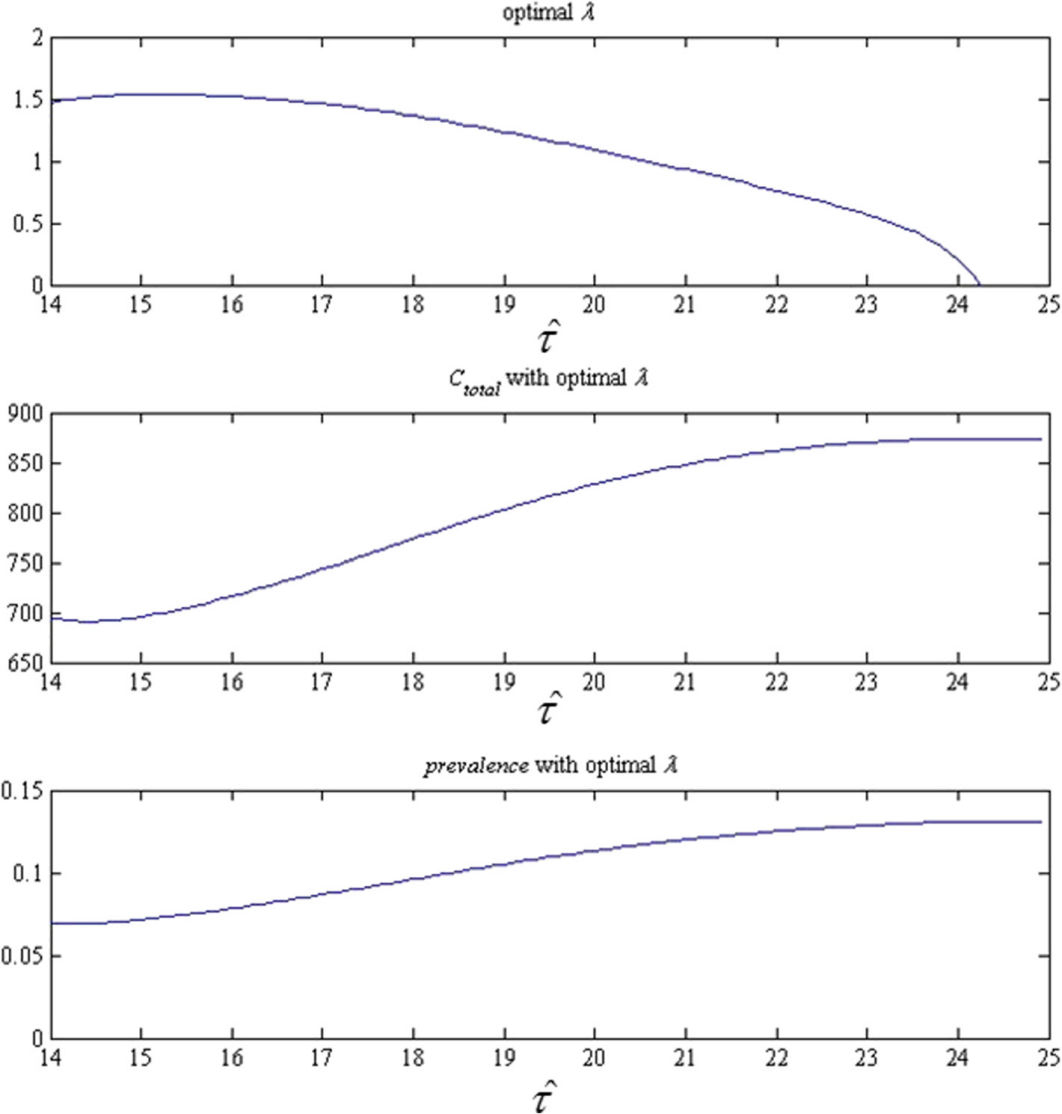
Screening rate, per-capita cumulative cost, and terminal prevalence of strategy S2 for each possible screening initiation age
- For S3, we present the optimal screening rates with all possible screening initiation ages, as well as the associated per-capita cumulative cost and terminal prevalence in Fig. 6. The strategy with the minimum cost is the one that starts the screening for every individual when she reaches the 4^th^ month after the 14^th^ birthday. The screening rate is 1.499 times per year.

**Fig. 6:**
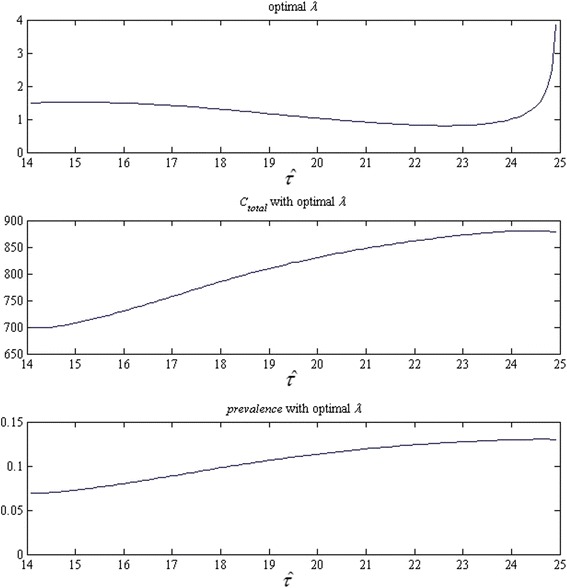
Screening rate, per-capita cumulative cost, and terminal prevalence of strategy S3 for each possible screening initiation age
- In Table 3, we compare the three strategies. First, the three studied strategies all outperform the strategy of no screening and the current CRC recommendations in both per-capita cumulative cost and terminal CT prevalence. Second, the comparison indicates the superiority of age-dependent CT screening strategy (S1 vs. S2) and quantifies its potential impact to the screening practice. Finally, the comparison shows comparable solution qualities between S2 and S3, suggesting the strategy design may not be sensitive to the quantification of age-dependent CT infection risks. In terms of computation time, on a PC with a 2.33GHz Intel Core 2 Duo Processor and 2GB RAM, the computation time is about 3.5 s for identifying S3, compared to 13 s for S2. This is mainly due to the fact that the gradient is available to the one-dimensional linear search for S3 but not for S2.

**Table 3:** Comparison between the screening strategies

Screening strategies	Per-capita cumulative cost	Terminal prevalence
No screening	$874	13.12 %
CDC Recommendations	$706	8.15 %
S1 w/ yearly update	$675.4	6.75 %
S1 w/ monthly update	$673.0	6.69 %
S2	$691.1	6.94 %
S3	$691.7	6.92 %

### Discussion

Overall, our numerical studies suggest that considering age-dependency in the screening strategy design is more cost-saving than currently recommended strategies. Our results further offer insights into various aspects of the design. With the study on S1, the results suggest that the age-dependency on the screening rate in an optimal screening policy roughly coincides with the age-dependency on the CT infection risk. That is, the screening rate should be intensified around age 16 – 18, which is the age range where the infection risk is highest. Compared to the current recommendations, biannual screening or screening every 8 months is more likely to be optimal from the societal cost-saving viewpoint. With the study on S2, the results suggest that it may be beneficial to initiate the screening earlier at least for the tested intercity cohort, which has relatively high CT prevalence. This also suggests that it is important to consider the potential costs incurred by the PID sequelae. Thus, it is important to provide accurate estimate on the probabilities of developing the sequelae in any strategy design activities.

Comparing S2 to S1 suggests that constant rate screening is likely to be acceptable given the small increase in both outcomes. Comparing S3 to S2 suggests that accurate quantification of age-specific CT infection risks may not be essential to the design of strategies with constant screening rate. Note that almost all the existing work largely relies on relatively crude estimates due to data scarcity and ethical concerns [31]. Finally, the fast computations suggest that it may be appealing to expand our models to incorporate high-level population heterogeneities.

## Conclusions

In this research, we present a series of parameter optimization models to investigate age-dependent screening strategies for controlling chlamydia infection among young women. Through our modeling research, we attempt to inform the design of optimal population-based CT screening strategies from a societal cost-saving perspective while ensuring a sufficient level of practicality. For the analysis, we extend a widely used SEIRS model to incorporate age-dependent screening rate profile and apply a gradient-based line search algorithm for ease of numerical optimization.

Our future research will mainly be focused on detailed model development. For example, it is evident that risks of first-time infection and subsequent reinfection differ due to partial protective immunity against CT [11, 37]. We will formulate the parameter optimization models that differentiate individuals with first-time infection and reinfection. We will also consider different patterns in ongoing sexual partnership. We plan to adapt the pair compartment model in Heijne et al. [30], which captures sexual partnership duration and reinfection. The investigation on sexual partnership and effective management of sex partners motivates us to explore the use of stochastic network models (e.g., [5, 32]), which provides added flexibility in modeling sexual partnership networks of complex structure. We will thereby develop optimization models based on stochastic network models for CT transmission among heterogeneous sex partners. In addition, we will model programmatic adherence and testing accuracy to make our strategy design more suitable in real-world CT infection control. Other future research directions include design of more efficient parameter optimization solution methods, systematic literature review for model parameter estimation, and sensitivity analyses on the model parameters.
